# Population-level analysis of glycoprotein glycoforms

**DOI:** 10.1080/19420862.2026.2665879

**Published:** 2026-04-30

**Authors:** Alejandro Gomez Toledo, James T. Sorrentino, Sanne Schoffelen, Bjørn Voldborg, Erika Velasquez, Aaron M. Scott, Göran Larson, Nathan E. Lewis, Johan Malmström

**Affiliations:** a Division of Infection Medicine, Department of Clinical Sciences, Lund University, Lund, Sweden; b Bioinformatics and Systems Biology Graduate Program, University of California, San Diego, La Jolla, CA, USA; c Department of Biotechnology and Biomedicine, Technical University of Denmark, Kgs. Lyngby, Denmark; d IPSC Laboratory for CNS Disease Modelling, Department of Experimental Medical Science, BMC D10, Lund University, Lund, Sweden; e Department of Laboratory Medicine, Institute of Biomedicine, University of Gothenburg, Gothenburg, Sweden; f Department of Clinical Chemistry, Region Västra Götaland, Sahlgrenska University Hospital, Gothenburg, Sweden; g Departments of Pediatrics and Bioengineering, University of California, San Diego, La Jolla, CA, USA; h Complex Carbohydrate Research Center, University of Georgia, Athens, GA, USA; i Department of Biochemistry and Molecular Biology, University of Georgia, Athens, GA, USA; j Center for Molecular Medicine, University of Georgia, Athens, GA, USA

**Keywords:** Glycan heterogeneity, glycoproteomics, glycosylation, immunoglobulin

## Abstract

The structure and function of many proteins are regulated post-translationally through glycan attachment. These glycans, assembled via competing enzymatic reactions, generate diverse glycoform populations – variants sharing a protein backbone but differing in glycan structures. While current analyses often focus on individual glycoforms, we demonstrate that population-level glycoform analysis – integrating spectral, biosynthetic, and physicochemical relationships – reveals new insights into glycoprotein regulation. Applied to immunoglobulin subclasses and antithrombin III (AT3), this approach provides comprehensive coverage of glycoform repertoires from human and murine plasma and biopharmaceuticals. It also enables sensitive quantification of glycosylation changes arising from in vitro manipulations or in vivo infections. Finally, we introduce a statistical framework adapted from ecological biodiversity studies, revealing that both IgG and AT3 exhibit skewed glycoform distributions shaped by biosynthetic constraints and degradation. Our findings demonstrate the added value of population-level glycoform analysis in understanding protein function and regulation through glycosylation.

## Introduction

Mammalian genomes encode a limited number of genes, but generate a much greater diversity of proteins through genetic mechanisms and post-translational modifications (PTMs). Among PTMs, glycosylation is the most chemically and structurally complex. Protein glycosylation involves the attachment of glycan chains to specific sites on proteins, which influences their structure, function, and interactions with other biomolecules.^1^ For example, glycans stabilize the crystallizable fragment (Fc) of mammalian antibodies, modulating interactions with immune cell receptors that trigger protective functions such as phagocytosis and degranulation.^2–4^ Beyond antibodies, most mammalian membrane and secreted proteins, including receptors, hormones, growth factors, and other drug targets, are glycoproteins, underscoring the importance of glycosylation in medical and biotechnological applications.^5,6^

Unlike proteins, which are genome-encoded, cellular glycans are the product of competing enzymatic reactions that modify glycoprotein substrates as they pass through the secretory pathway. As a result, a glycoprotein is never a single entity, but a population of related glycoforms, i.e., proteins with identical amino acid sequences but distinct glycan chains attached at specific sites.^7^ The abundance and diversity of these site-specific glycoforms are influenced by factors such as enzyme abundance and specificity, subcellular localization and micromilieu, and access to donor molecules and glycosylation sites.^8–10^ Altered glycoform distributions are linked to inherited metabolic diseases, such as congenital disorders of glycosylation, or to acquired diseases affecting cellular metabolism, including cancer and infections.^11^ Furthermore, pathogenic microbes may express glycosidases that degrade host glycoforms, enabling tissue invasion and immune evasion.^12–14^ Given the critical role of glycoprotein glycoforms in health and disease and their potential as biomarkers and therapeutic targets, robust methods for their characterization and quantification are essential.

Mass spectrometry (MS)-based glycoproteomics is the preferred method for high-throughput characterization of glycoprotein glycoforms.^15,16^ In a typical workflow, glycoproteins are digested into peptides and glycopeptides, separated chromatographically, and analyzed by MS. In contrast to unmodified peptides, glycopeptides derived from site-specific glycoforms, which originate from common biosynthetic pathways, share structural and physicochemical properties such as size, hydrophobicity indices, and isoelectric points, making this analysis particularly challenging. Site-specific glycoforms frequently co-elute during reverse-phase chromatography – the most common separation method – and may also be co-isolated during MS acquisition, leading to chimeric spectra that are difficult to interpret.^17^ Additionally, the wide dynamic range of glycoform abundance, with some forms being highly prevalent and others present at low levels, further complicates this analysis. Notable structural and chromatographic differences can also arise when glycoforms carry charged monosaccharides or branching structures, or when a glycosylation site is only partially glycosylated.

Over the past few decades, glycoproteomics has advanced the development of various liquid chromatography (LC)-MS/MS strategies leveraging both data-dependent (DDA) and data-independent (DIA) MS acquisition methods to more efficiently tackle some of the challenges associated with glycoform analysis. DIA methods, in particular, provide a more quantitative framework for glycopeptide analysis, especially when combined with new robust bioinformatic pipelines that enable precise quantification of glycoform profiles across biological systems.^18–23^ Despite these advances, MS-based glycoform analysis is still laborious and technically demanding. Consequently, the glycoform landscape of biologically significant glycoproteins, such as immunoglobulin G (IgG) antibodies – key players in adaptive immunity and a rapidly growing class of biopharmaceuticals – remains incompletely understood. IgG glycosylation varies across subclasses (e.g., human IgG1-4; mouse IgG1-3) and is influenced by disease conditions and aging.^24,25^ Previous studies have identified ~20–30 IgG glycoforms circulating in human and mouse plasma,^24,26,27^ but it remains unclear whether this reflects a natural biosynthetic limit or insufficient sensitivity in current analytical methods.

The objective of this study was to develop an experimental and computational framework that complements existing glycoproteomic strategies by analyzing glycoproteins not as isolated ‘single’ molecules, but as glycoform populations that share common structural and physicochemical features. This perspective is both intuitive and practical, as changes in glycosylation – whether due to variations in glycosyltransferase activity or the availability of sugar nucleotides – do not merely affect individual glycoforms but propagate across the entire glycoform population processed by these enzymes. Additionally, because site-specific glycoforms share a common protein backbone, any transcriptional or translational changes affecting the core protein will affect the entire glycoform population. Here, we introduce a population-level analytical framework to study populations of glycoprotein glycoforms and demonstrate that this approach is well suited to: 1) interrogate the total spectrum of glycoforms at a given glycosylation site, 2) analyze their collective abundance distributions, and 3) assess their coordinated responses to both intrinsic and extrinsic perturbations. This methodology complements current glycoproteomic strategies by enhancing structural coverage of glycoform populations while improving both analytical sensitivity and quantification.

## Results

### Outline of the population-level analysis

**Figure 1.**
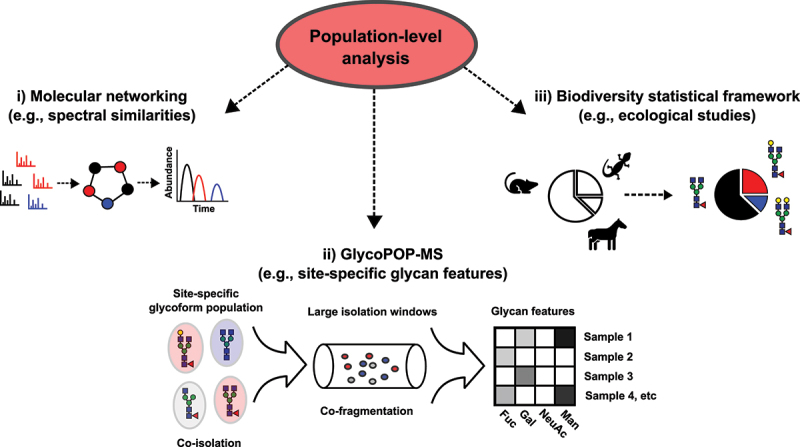
Population-based analytical approach. Schematic outline of the approach highlighting the three main population-level analytical components: (i) molecular networking in combination with a network walk algorithm for improved node annotation, ii) the GlycoPOP-MS experimental approach, and (iii) a statistical biodiversity framework.

To facilitate population-level analysis of glycoforms, we first developed an analytical strategy that integrates spectral, biosynthetic, and physicochemical relationships across site-specific glycoform populations. This strategy consists of three interconnected components (Figure 1):

1. Molecular networking: a bioinformatic approach that clusters glycopeptide MS/MS data into networks based on spectral similarities. The glycopeptide networks are subsequently annotated with information derived from database searches, and complemented with expanded annotations via a “network walk,” a computational algorithm that transfers annotations between clustered neighboring nodes. These expanded glycopeptide networks are then converted into glycopeptide spectral libraries containing transition lists to quantify glycopeptide precursor abundances.
2. GlycoPOP-MS: a DIA-MS method designed to directly co-isolate and co-fragment site-specific glycoforms. The resulting DIA fragmentation patterns are mined to generate transition lists associated with population-level glycan features, and these GlycoPOP-MS transitions are stored as spectral libraries to enable their quantification. Compared to traditional DIA-MS glycoproteomics workflows, which generally aim to assign peak groups back to individual precursor glycopeptides, GlycoPOP-MS is specifically designed to capture site-specific glycoform populations as a whole. To achieve this, it uses substantially broader isolation windows (up to 600 Da) to intentionally capture all co-eluting glycopeptides that share the same peptide backbone but differ in glycan composition. Rather than deconvoluting fragments at the level of individual precursors, as in regular DIA workflows, GlycoPOP-MS focuses on extracting glycan-specific population-level features, such as the total abundance of galactose, fucose, or sialic acid associated with a given glycosylation site across the full glycoform population. This population-centric strategy improves sensitivity and provides a more direct readout of biologically relevant glycan traits.
3. A Biodiversity statistical framework: a mathematical framework adapted from ecological studies to quantify the statistical structure of glycoform populations. This framework provides insights into the abundance distribution patterns and dynamic changes in glycoform populations in response to biosynthetic perturbations and infections.

Our workflow, which is based on a population-level analysis combining these three components, enables one of the most exhaustive inventories of the IgG glycoform landscape to date, including its abundance distribution patterns and dynamic responses to genetic manipulation and environmental stimuli (Supplementary Figure S1).

### Mapping the IgG glycoform landscape

First, we tested the performance of our population-level analysis to exhaustively capture the glycoform landscape associated with human IgG. IgG antibodies have been the focus of many glycoproteomic studies and their glycosylation patterns are relatively well understood. However, the range of glycans that can be accommodated on the IgG Fc region remain incompletely mapped. We analyzed human IgG from various biological sources using a state-of-the art LC-MS/MS glycoproteomics workflow based on DDA acquisition and stepped higher energy collisional dissociation (HCD) fragmentation, to generate a representative MS spectral library capturing a broad spectrum of IgG glycoforms. The samples included: 1) commercial intravenous Immunoglobulin (IVIG) preparations and IgG isolated from pooled human plasma, both reflecting the native IgG glycan patterns found in healthy individuals; 2) four commercial monoclonal antibodies: Avastin® (AVA; bevacizumab), Erbitux® (ERB; cetuximab), Herceptin® (HER; trastuzumab), and Xolair® (XOL; omalizumab), exhibiting heterogeneous glycan profiles, including glycans not normally found on native IgG; and 3) four monoclonal antibodies produced in glycoengineered Chinese hamster ovary (geCHO) cells, designed to express glycoproteins with less core fucosylation (FUT), less antennae galactosylation (GAL), or more α2,6-sialylation (ST), compared to the wildtype CHO cells (WT).^28^ Additionally, some of the monoclonals were enzymatically pretreated with sialidases and galactosidases to increase structural diversity.

All antibodies were digested with trypsin, and the peptide digests were separated by standard reverse-phase chromatography and analyzed by LC-MS/MS. The antibody samples were analyzed in 40 MS injections, and all MS raw files were searched against a human protein and glycan database (Supplementary data 1) using Byonic with previously optimized search parameters.^29^ In total, we identified 184 unique glycan and peptide combinations from tryptic and semitryptic glycopeptides derived from human IgG1-4 (Supplementary data 2). The glycoform distributions were: IgG1 (37 glycoforms), IgG2 (26 glycoforms), and IgG3/4 (22 glycoforms). The tryptic peptides from IgG3 and IgG4 are isobaric and thus here reported together as IgG3/4. Both the number and type of structural variants were in line with previous reports.^30^

### Molecular networking expands the number of identifiable IgG glycoforms

We previously leveraged spectral similarities to cluster and organize glycopeptide MS/MS data, facilitating the identification of new glycoforms, including unexpected glycan modifications.^31–33^ Here, we expanded our scope and tested whether this approach could facilitate not only identification but also quantification of new glycoforms, through generation of a large spectral library of IgG glycoforms. To accomplish that, we combined all MS/MS scans containing glycan-specific oxonium ions (e.g., m/z 204.09 Da, GlcNAc) across all samples and subjected them to spectral clustering. The resulting glycopeptide networks were then annotated with the Byonic search results, yielding partially annotated networks. Additionally, we developed a “network walk” algorithm that propagates annotations from fully identified nodes to unannotated nodes. This propagation is based on precursor mass shift intervals that correspond to monosaccharide mass differences between neighboring nodes, which were initially clustered due to spectral similarities (see Material and Methods section).

**Figure 2.**
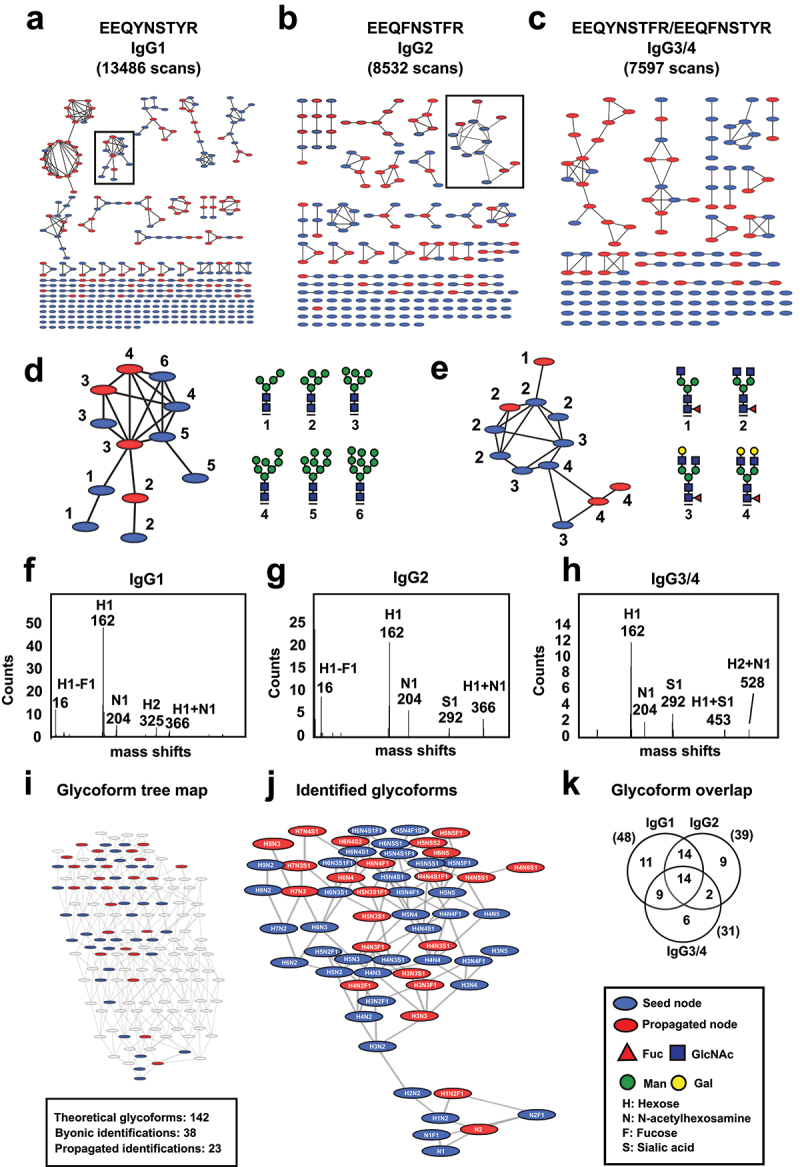
Glycoform landscape of human IgG. Molecular networking analysis of glycopeptide spectra derived from (a) IgG1, (b) IgG2, and (c) IgG3/4, organized by spectral similarity. Initial node annotation was based on results from Byonic database searches (blue nodes), followed by propagation through the network walk algorithm (red nodes). (d) Example cluster of IgG1 glycopeptides showing high-mannose structures (insert in a). (e) IgG2 glycopeptide cluster with complex-type N-linked glycans (insert in B). Frequency plots showing precursor m/z differences across the (f) IgG1, (g) IgG2, and (h) IgG3 networks. (i) Tree map displaying theoretical glycoforms inferred from identified glycopeptides alongside experimentally observed glycoforms. (j) Summary of all identified IgG glycoforms. (k) Venn diagrams illustrating identified glycoforms across IgG subclasses.

As we have shown previously, only a fraction of the nodes of these glycopeptide networks can be annotated^32^; however, our approach was still able to match 13,486 scans to 376 nodes for IgG1, 8532 scans to 201 nodes for IgG2, and 7597 scans to 138 nodes for IgG3/4 (Figure 2(a-c) and Supplementary data 3). An example of an annotated cluster from the IgG1 network is shown in Figure 2(d), representing a fully tryptic glycopeptide with various high-mannose N-linked glycans. These glycoforms mainly differed in the number of mannose residues, which ranged from 4 to 9 monosaccharide units (Man-4 to Man-9). Another cluster from the IgG2 network is shown in Figure 2(e), containing glycopeptides carrying bi-antennary complex-type N-linked glycans with varying terminal GlcNAc and Gal modifications. In all cases, several spectra that remained unassigned after the initial Byonic searches were annotated through the “network walk” (IgG1: +5444 scans, IgG2: +2573 scans and IgG3/4: +3664 scans), revealing glycopeptides with different proposed compositions, charge states or modified with chemical adducts. Precursor mass differences between neighboring nodes across the entire networks indicated that most variations within each glycoform population could be attributed to specific monosaccharide and disaccharide differences (Figure 2(f-h)).

Finally, we extracted spectral information from all identified glycoforms across IgG subclasses and used GlyCompare ^34^ to generate a complete glycoform network, incorporating both observed glycoforms and biosynthetically inferred intermediates based on N-linked glycosylation rules (Figure 2(i) and Supplementary data 4). The total number of theoretical glycoforms was 142, of which 38 were experimentally identified via database searches, with an additional 23 identified through the “network walk” (Figure 2(j) and supplementary FigS2). This analysis resulted in an increase in the number of unique glycoforms for each IgG subclass: IgG1 (from 37 to 48), IgG2 (from 26 to 39), and IgG3/4 (from 22 to 31) with significant overlap between subclasses (Figure 2(k)). Among these additional glycoforms, we found high-mannose, hybrid and sialylated glycans, which are sparsely observed in human plasma using regular glycoproteomic analysis. Taken together, this analysis shows that a population-level approach, combining molecular networking and a “network walk” algorithm can expand the number of identifiable IgG glycoforms by leveraging spectral relationships. However, the number of identifiable glycoforms is still only a fraction of all theoretically possible ones, suggesting that biosynthetic constraints are likely shaping the output of the cellular glycosylation machinery.

### Quantifying IgG glycoform diversity

**Figure 3.**
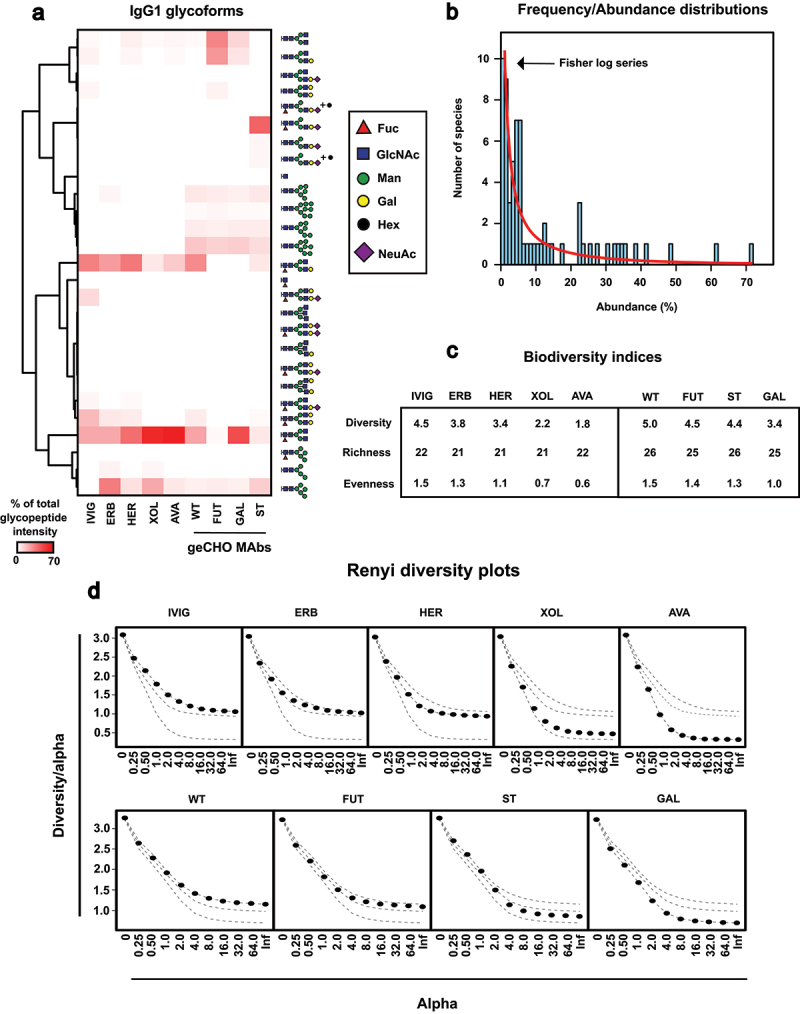
Quantification of IgG glycoform distributions using a biodiversity framework. (a) Relative quantification of IgG1 glycoforms across commercial monoclonal antibodies and IgG antibodies expressed in geCHO cell samples. (b) Species frequency/abundance distributions across all quantified IgG glycoforms, modeled with Fisher’s log series (red line). (c) Biodiversity indices based on glycoform heterogeneity. (d) Renyi diversity plots estimating the heterogeneity of IgG1 glycoform populations. Experiments were performed in three technical replicates (e.g., independent enzyme digestions) and the average values are plotted. Abbreviations: ERB: Erbitux, her: herceptin, XOL: xolair, AVA: Avastin, wt: wildtype, fut: FUT8 knockout, st: ST6GAL1 knock-in, GAL: B4GALT1 knockout.

Previous studies have indicated that not all identifiable site-specific glycoforms are equally abundant, and their abundance distribution patterns are sensitive to biological context.^7^ Therefore, we used the spectral libraries obtained through networking as a starting point to quantify the IgG glycoform diversity in each individual sample. To accomplish that, we generated transition lists for all identified glycopeptide precursors and used their associated MS1 precursor signals to quantify the abundance of individual IgG1 glycoforms using Skyline, an established bioinformatic tool for quantitative proteomics.^7^ Of the 38 observed glycoforms, only 27 had signal-to-noise ratios above the quantification threshold and were quantifiable across any of the conditions (Figure 3(a), Supplementary data 5).

Both commercial and glycoengineered monoclonal antibodies exhibited very different glycoform profiles compared to the physiologically relevant glycosylation found in IVIG (Figure 3(a)). For instance, the IVIG glycoform population displayed a higher degree of galactosylation and sialylation than the monoclonal antibodies, clearly highlighting the challenges in reproducing endogenous IgG glycosylation from B cells using recombinant expression systems. As expected, high-mannose glycans, which typically have very short half-lives in plasma,^35,36^ were barely detected in IVIG, but featured prominently in all recombinant antibodies, with Man-5 being the most abundant glycoform. Additionally, the glycoengineered antibodies contained quantifiable amounts of larger high-mannose N-linked glycans (Man-6 to Man-9).

The glycoform profile of the monoclonal antibodies expressed in geCHO cells was consistent with the expected outcomes of their specific genetic manipulations. For example, α- ^1,6^-fucosyltransferase (FUT8) mutants produced IgG1 completely devoid of core fucosylation, while β-1,4-galactosyltransferase 1 (B4GALT1) mutants produced IgG1 lacking any trace of galactosylation. Similarly, mutant knock-in cells expressing sialyltransferase β-galactoside alpha-2,6-sialyltransferase 1 (ST6GAL1) produced more sialylated IgG1 compared to wild-type CHO cells. However, the most abundant sialylated glycoform, the mono-antennary HexNAc ^3^Hex ^4^Fuc ^1^NeuAc,^1^ differed significantly from the structure of the most abundant sialylated IgG1 glycoform found in IVIG, which was the bi-antennary HexNAc ^4^Hex ^5^Fuc ^1^NeuAc.^1^

### A biodiversity statistical framework captures IgG glycoform diversity

While almost all glycoproteomic studies consistently find that glycoforms vary in abundance, only a few have attempted to enumerate site-specific glycoform diversity.^7,37,38^ Similarly, we found that, despite differences in IgG1 glycoform profiles resulting from variations in expression systems and biosynthetic manipulations, the abundance distributions remained skewed toward a few dominant glycoforms (Figure 3(a)). To better understand the statistical structure of these glycoform distributions, we recognized that the problem is similar to measuring biological diversity in ecological studies.^39^ In ecology, many statistical approaches and concepts, such as species frequency/abundance distribution plots and biodiversity indices, have been developed to capture the variation in heterogenous species populations and could be applied here.^40,41^

Using species frequency/abundance distribution plots derived from biodiversity studies, we found that the IgG1 glycoform distributions in our dataset followed a logarithmic distribution, which could be modeled using a Fisher’s log series, often used to describe populations with a few common species and many rare ones (Figure 3(b)). This is consistent with a glycoprotein population with only a few highly abundant glycoforms, while the vast majority are rare or infrequent. We also modeled the species frequency/abundance distribution to two other statistical models of biodiversity given similar results (Supplementary Figure S2b). To further quantify this glycoform diversity, we calculated a set of basic biodiversity indices, including richness (the total number of distinct glycoforms), evenness (how uniformly glycoform abundances are distributed across the population), and the inverse Simpson index, a diversity index that accounts for both the abundance of dominant glycoforms and the presence of rare ones. These indices complement the Fisher log series by providing a more nuanced view of glycoform diversity (Figure 3(c)). For example, the IVIG samples exhibited the highest glycoform diversity based on the inverse Simpson index (4.5), while AVA showed the lowest diversity (1.8). Interestingly, there was no difference in the number of distinct glycoforms (richness) between the two, but the main variation lay in evenness. IVIG glycoforms were slightly more spread out in terms of their abundances, with an evenness value of 1.5, whereas the glycoform abundances of AVA were more skewed, with an evenness of just 0.6. This indicates that while both samples had similar numbers of glycoforms, the distribution of their abundances was much more uniform in IVIG compared to AVA.

Interestingly, when measuring biodiversity indices for the glycoengineered monoclonal antibodies, we found that the B4GALT1 mutant exhibited the lowest glycoform diversity, with an inverse Simpson index of 3.4 and an evenness of 1.0, compared to the wild-type antibody, which had higher diversity (5.0) and evenness (1.5). This is consistent with galactose being required for the addition of terminal sialic acids, and the absence of galactose in the B4GALT1 mutant had a more profound effect on the glycoform population, leading to a more skewed glycoform profile.

Finally, we also modeled glycoform diversity using Renyi plots. A Renyi plot provides a more complete view of diversity by showing how diversity indices change at different scales of abundance. It plots the diversity orders (or alpha values) against the corresponding diversity measures, offering a way to compare both the richness and evenness across different samples. For example, we uncovered a marked difference between the Renyi profiles of IVIG, ERB, and HER, which exhibited higher glycoform diversity than XOL and AVA (Figure 3(d)). The Renyi plots further clarified the pattern for monoclonal antibodies from the geCHO cells, with the B4GALT1 mutant showing the lowest diversity, in line with the absence of galactose and its significant impact on glycoform distribution. In summary, our findings highlight the complexity of IgG1 glycoform diversity and demonstrate how statistical tools from ecology, such as biodiversity indices and Renyi plots, might be effective in revealing hidden patterns in glycoform diversity across different samples.

### Evaluating the glycoform diversity of antithrombin 3

**Figure 4.**
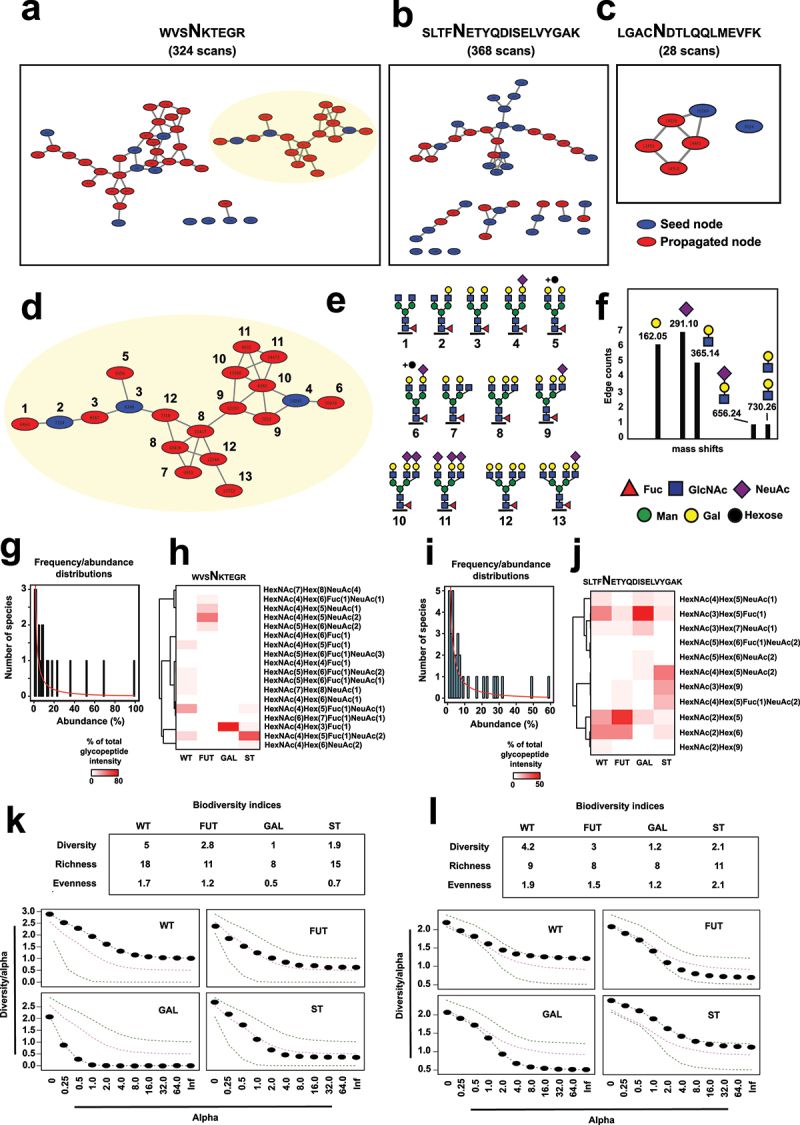
Site-specific glycosylation analysis of antithrombin 3 (AT3). Networking analysis of glycoform populations associated with the N-linked glycosylation sites within the peptide sequences: (a) WVSNKTEGR, (b) SLTFNETYQDISELVYGAK and (c) LGACNDTLQQLMEVFK. Blue nodes: seed nodes identified through the Byonic searches; red nodes: propagated nodes through the network walk. (d) Example glycopeptide cluster from a and (e) the cartoon representation of the specific glycoforms associated with each node in e and numbered accordingly. (f) Frequency plots showing precursor m/z differences across the example cluster in d. (g) Frequency/abundance/abundance distribution plots and (h) relative quantification of the glycoforms associated with site WVSNKTEGR and quantified using relative precursor intensities as percentages of the total glycopeptide intensity of the glycoform population. (i) Frequency/abundance/abundance distribution plots and (j) relative quantification of the glycoforms associated with site SLTFNETYQDISELVYGAK and quantified using relative precursor intensities as percentages of the total glycopeptide intensity of the glycoform population. Biodiversity indices (top) and Renyi plots (bottom) for site WVSNKTEGR (k) and site SLTFNETYQDISELVYGAK (l). Experiments were performed in three technical replicates (e.g., independent enzyme digestions) and the average values are plotted. Abbreviations: wt: wildtype, fut: FUT8 knockout, st: ST6GAL1 knock-in, GAL: B4GALT1 knockout.

Next, we tested whether this type of population-based approach could be extended to more complex cases, such as glycoproteins with more elaborate glycans and multiple glycosylation sites. To do this, we expressed human antithrombin 3 (AT3) in the same panel of geCHO cells. AT3 is a glycoprotein regulator of the coagulation pathway and an important therapeutic target.^42^ It has four potential N-glycosylation sites, three of which were identified in these samples through initial DDA LC-MS/MS analysis, carrying a wide range of glycoforms (Supplementary data 6). In total, 49 unique glycopeptides (e.g., unique combinations of peptide + glycan) were identified across these sites, corresponding to 6 glycoforms associated with site: LGACNDTLQQLMEVFK, 28 glycoforms associated with site: SLTFNETYQDISELVYGAK, and 11 glycoforms associated with site: WVSNKTEGR. The MS/MS data was also subjected to the same networking workflow used for IgG, which resulted in the identification of several clusters of related glycoform spectra associated with each of these three glycosylation sites (Figure 4(a-c)).

In total, 41 nodes were directly annotated using results from the Byonic searches, while 66 additional new nodes were identified through the network walk. We focused on the glycosylation patterns associated with the acceptor sites WVSNKTEGR and SLTFNETYQDISELVYGAK since they were strikingly heterogeneous, with significant differences in their levels of galactosylation, sialylation, high-mannose content, and branching patterns. As shown in Figure 4(d-f) and Supplementary data 7, the network walk was effective at identifying tri- and tetra-antennary glycan structures, which were missed by the Byonic searches, despite those proposed structures being included in the original search databases. A total of 36 unique glycoforms were identified at the WVSNKTEGR site, 18 of which were quantifiable in Skyline using a transition list containing these precursors. Similarly, 26 unique proposed structures were identified at the SLTFNETYQDISELVYGAK site, with 11 being quantifiable. In general, the glycoform landscape produced by the geCHO cells aligned well with the expected structural output of each genetic manipulation (Figure 4(g-j)).

Frequency and abundance distribution plots of the glycan diversity at each site revealed skewed glycan distributions that could be modeled using Fisher’s log series, as previously also observed for IgG glycans, suggesting that this might be a common feature of site-specific protein glycosylation (Figure 4(g) and (i)). We calculated diversity indices for each site, which indicated that, in both cases, AT3 produced in glycosylation mutant geCHO cells displayed lower overall diversity compared to wildtype samples, as indicated by lower inverse Simpson indices (Figure 4(k-l), top). Both richness and evenness contributed to the overall diversity differences at the WVSNKTEGR site, with B4GALT1-knockout cells producing the least diverse AT3 glycoform populations. A similar reduction in diversity was observed at the SLTFNETYQDISELVYGAK site, where galactose-deficient glycoforms were the least diverse. However, Renyi diversity plots showed that the introduction of ST6GAL1 (ST samples) considerably reduced the overall diversity profile at the WVSNKTEGR site, but not at the SLTFNETYQDISELVYGAK site, compared to wildtype CHO AT3 (Figure 4(k-l), bottom). This indicates that site-specific glycan diversity is shaped by the expression of glycosyltransferases, as well the nature of the specific acceptor sites.

### Measuring glycoform populations through GlycoPOP-MS

As demonstrated above, deep characterization and quantification of glycoform populations can be achieved through bioinformatic and statistical analyses that recognize structural similarities between glycoforms. However, these analyses still rely on the experimental acquisition of individual data points derived from specific glycoforms. Instead of isolating individual glycoforms, we hypothesized that capturing the entire co-eluting glycoform population simultaneously could generate fragmentation spectra encompassing all glycan variations within the population. To achieve this, we developed GlycoPOP-MS, A DIA-based acquisition workflow based on broad isolation windows to co-fragment co-eluting glycoform populations.

**Figure 5.**
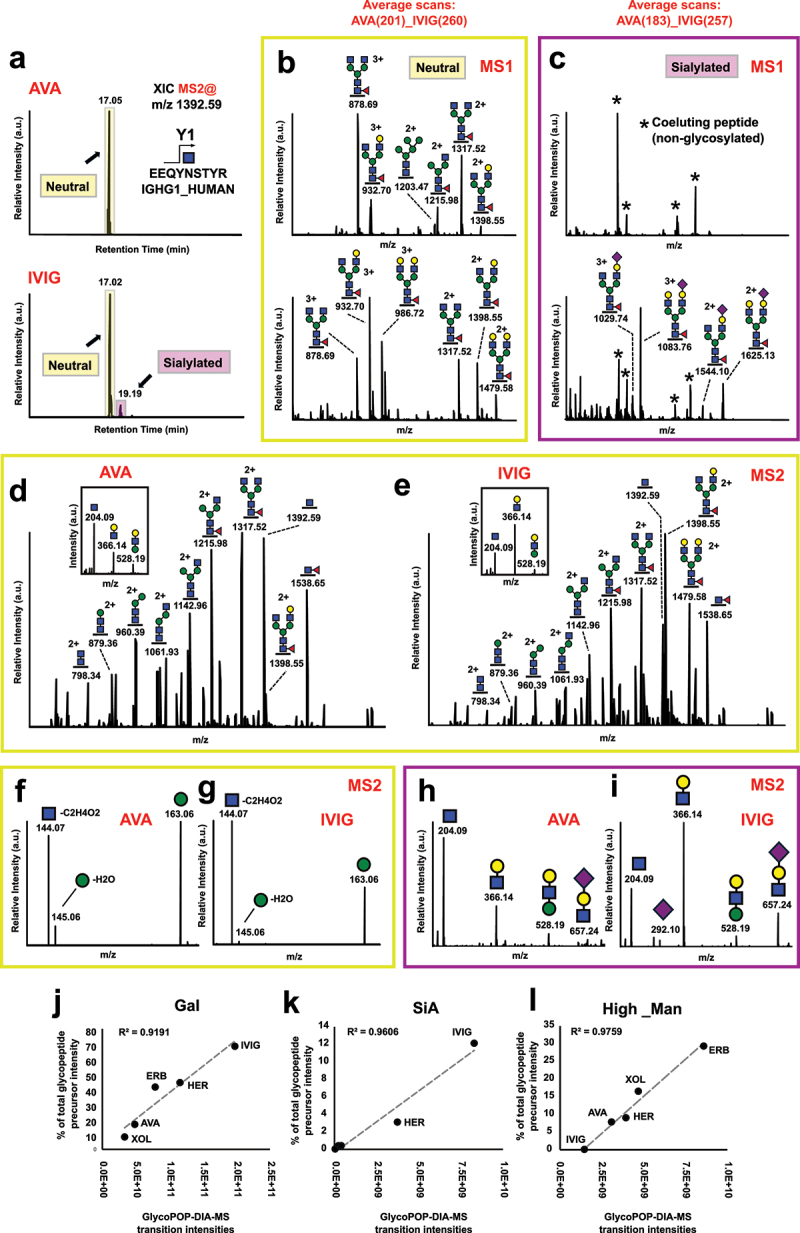
GlycoPOP-MS glycoform quantification. (a) Comparison of co-eluting neutral and sialylated IgG1 glycopeptide precursors from Avastin (AVA, top) and IVIG (bottom) samples. Average MS1 profile of MS1 precursors eluting in (b) the neutral and (c) sialylated retention time windows shown separately from AVA (top) and IVIG (bottom). Average GlycoPOP-MS fragmentation spectra over the neutral glycopeptide time window for (d) AVA and (e) IVIG over the chromatographic window where neutral glycopeptide elute. Average GlycoPOP-MS fragmentation signature for oxonium ion transitions that are indicative of high-mannose structures (e.g., m/z 145.06 and m/z 163.06) in (f) AVA and (g) IVIG samples. Average GlycoPOP-MS fragmentation signature for oxonium ion transitions that are indicative of sialylated structures (e.g., m/z 292.10 and m/z 657.24) in (h) AVA and (i) IVIG samples. GlycoPOP-MS quantification of IgG1 (j) galactose, (k) sialylation, and (l) high-mannose levels correlates with measurements of monosaccharide contents through integration of precursor MS1 signal expressed as a percentage of total glycopeptide intensity. Experiments were performed in three technical replicates (e.g., independent enzyme digestions) and the average values are plotted. Abbreviations: ERB: Erbitux, her: herceptin, XOL: xolair, AVA: avastin.

To optimize the GlycoPOP-MS workflow, we first analyzed IgG1 glycopeptides from AVA and IVIG samples, which differ significantly in their glycoform distributions (Figure 3). As shown in Figure 5(a-c), tryptic IgG1 glycopeptides from both samples share the same peptide backbone and co-elute during reversed-phase chromatography. In AVA samples, all glycopeptides carrying neutral glycans co-elute around 17 minutes (Figure 5(a), top), while IVIG samples show the same peak along with a later peak (~2 minutes later) corresponding to sialylated glycopeptides (Figure 5(a), bottom). The AVA peak contains glycoforms with lower galactosylation but higher abundance of high-mannose glycans compared to IVIG (Figure 5(b)). The sialylated glycopeptides in IVIG primarily consist of fucosylated and monosialylated glycoforms with one or two galactose residues (Figure 5(c), bottom). No sialylated precursors were detected in AVA (Figure 5(c), top).

Next, we monitored the intensity of the GlcNAc oxonium ion (m/z 204.09), a diagnostic marker for N-linked glycopeptides. Expanding the MS isolation window from ~3 Da (DDA) to 30 Da, 200 Da, and 600 Da significantly increased ion intensity due to co-isolated glycoform accumulation (Supplementary Figure S3a). The effect was most pronounced in the 200 Da and 600 Da windows, with the latter yielding a nearly two-fold increase compared to the former. We then examined the impact of isolation window size and collision energy on fragment transitions using narrow (30 Da) and broad (600 Da) DIA windows. Co-isolated precursors were fragmented at varying collision energies (15, 25, and 35%) and with a stepped HCD approach combining these energies. We generated theoretical fragment transitions covering expected oxonium ions and sequential monosaccharide losses from the identified precursors down to the Y1 ion. Using Skyline, we extracted 1,800 quantifiable features combining fragment type, energy, and isolation window size across AVA and IVIG samples (Supplementary data 8).

As previously shown for regular glycopeptide fragmentation, lower collisional energies favored monosaccharide neutral losses, while higher energies produced more oxonium ions and Y1 ions (Supplementary Figure S3b). Fragmentation patterns were unaffected by window size, but overall fragment intensities were significantly higher with the broad 600 Da window, consistent with the m/z 204.09 ion results. We conducted partial least squares discriminant analysis (PLS-DA) on this data set and identified the top 20 discriminative features that distinguished AVA from IVIG populations, which primarily corresponded to fragment transitions indicative of galactosylation, sialylation, and high-mannose structures (Figure 5(d-h), and Supplementary Figure S3c-e). These discriminant transitions were further experimentally validated by treating IVIG samples with galactosidases and sialidases, followed by GlycoPOP-MS analysis (Supplementary Figure S4).

Finally, we added an MS1 scanning dimension to the 600 Da DIA scheme for precursor quantification within the same experiment. Compared to conventional MS1 quantification using DDA LC-MS/MS, GlycoPOP-MS has shorter cycle times, yielding more data points across peaks, and resulting in more robust quantification with lower coefficients of variation (Supplementary Figure S5). Equipped with this optimized setup, we selected the most glycan-informative transitions (Supplementary table S1) and quantified galactosylation, sialylation, and high-mannose glycans across four commercial monoclonal antibodies and IVIG samples (Figure 5(j-l) and Supplementary data 9). GlycoPOP-MS quantification of galactose content closely mirrored MS1 precursor signal quantification (Figure 5(j)), and similar consistency was observed for sialylation (Figure 5(k)) and high-mannose glycans (Figure 5(l)). Notably, GlycoPOP-MS enabled quantification of low-abundance high-mannose glycans in IVIG that were undetectable in MS1. These results highlight the ability of GlycoPOP-MS to sensitively quantify trace glycosylation profiles in human antibodies.

### Population-level glycoform analysis of mouse IgG

**Figure 6.**
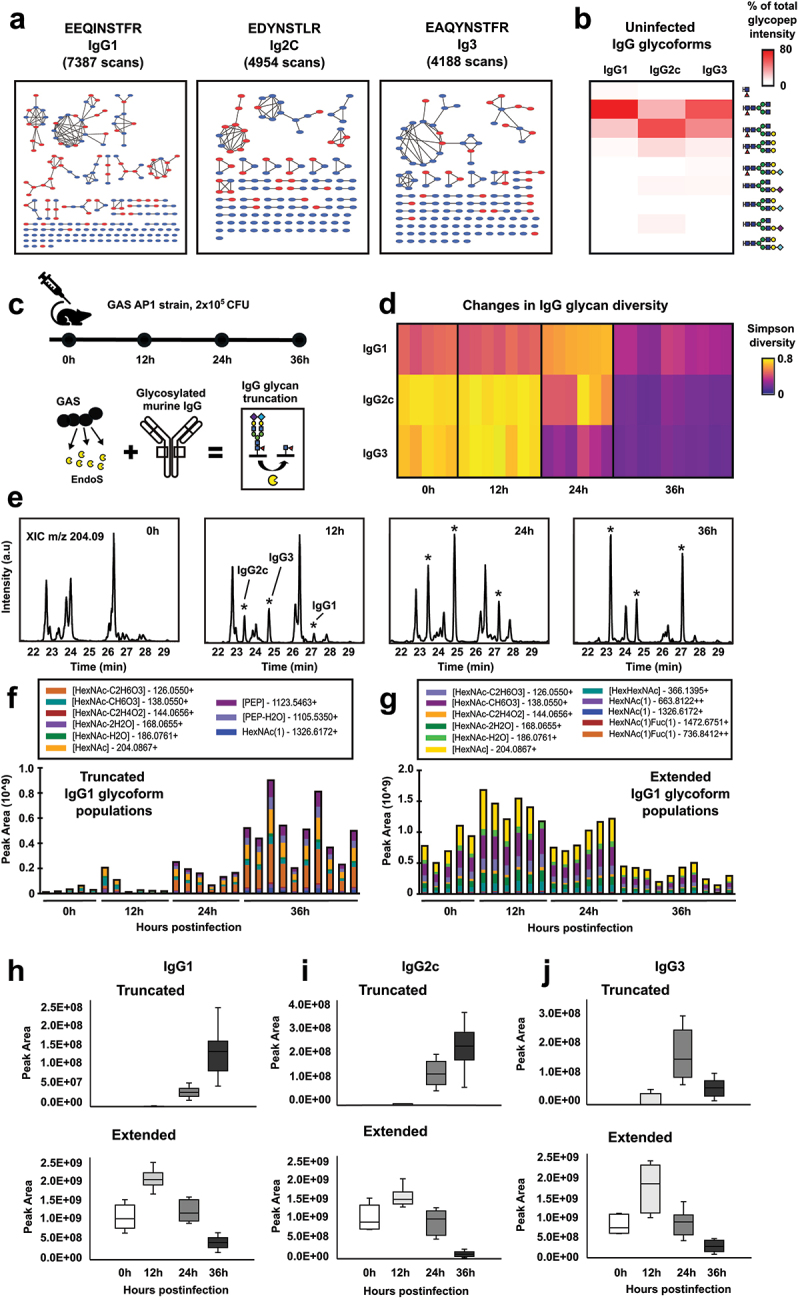
Population-based analysis of IgG glycosylation changes during murine gas infection. (a) Molecular network analysis of murine IgG from healthy mouse plasma (*n* = 5). Blue nodes: seed nodes identified through the Byonic searches; red nodes: propagated nodes through the network walk. (b) MS1 quantification of predominant IgG glycoforms using precursor intensities expressed as a percentage of the total glycopeptide intensity. (c) Infection workflow: mice were inoculated with gas, and plasma samples were collected at 12 h, 24 h and 36 h postinfection (*n* = 5–10 mice/condition) to evaluate IgG deglycosylation induced by bacterial EndoS. (d) Simpson diversity indices measuring changes in glycopeptide precursor MS1 intensities over the course of infection. (e) Extracted ion chromatograms for GlcNAc fragment ion m/z 204.09 across the infection timeline. GlycoPOP-MS quantification of (f) EndoS cleaved and (g) intact IgG glycoforms in blood samples from all mice from each time point. Each bar represents data obtained for individual mice. Integrated quantification changes of (h) IgG1, (i) IgG2C, and (j) IgG3 measured through GlycoPOP-MS using the transitions specified in the inserts of f and g. Upper whisker extends from the hinge to the largest value no further than 1.5 * IQR from the hinge (where IQR is the inter-quartile range). The lower whisker extends from the hinge to the smallest value, at most 1.5 * IQR of the hinge.

Building on the established population-level glycoform analysis for human antibodies, we applied this workflow to characterize the glycosylation landscape of murine IgG antibodies, given the central role of mice as preclinical models for human diseases, including infections and immune disorders. We isolated circulating antibodies from the plasma of healthy mice, digested them with trypsin, and subjected the samples to the same DDA LC-MS/MS workflow combined with molecular networking to generate a library of glycoforms. This analysis identified multiple glycoforms across three major murine IgG subclasses: IgG1, IgG2C, and IgG3, with nine glycoforms quantified with Skyline using MS1 precursor transitions (Figure 6(a-b) and Supplementary data 10). Similar to human IgG antibodies, the most prevalent murine Fc-glycoforms were complex-type N-linked glycans exhibiting varying degrees of galactosylation and sialylation, including terminal decorations with both N-acetylneuraminic acid (NeuAc) and N-glycolylneuraminic acid (NeuGc) (Figure 6(b)). Notably, IgG2C exhibited higher levels of galactosylation and sialylation than IgG1 and IgG3.

### Quantifying IgG glycosylation changes triggered by GAS infection

Previous research demonstrated that mice infected with group A streptococcus (GAS) progressively lose their IgG Fc glycans during infection,^12,43^ a process mediated by the bacterial enzyme EndoS, an IgG-specific endoglycosidase that targets the Fc region of both human and murine IgG antibodies. EndoS cleaves the β-1,4 linkage between the two GlcNAc residues in the chitobiose core of Fc glycans, leaving a single GlcNAc moiety attached to the protein, with or without core-fucosylation (Figure 6(c)). To evaluate the utility of a population-level glycoform analysis for tracking time-dependent IgG glycosylation changes during GAS infection, we subcutaneously inoculated mice with GAS and collected plasma at 12, 24, and 36 hours post-infection. IgG antibodies were then isolated and analyzed using GlycoPOP-MS.

Extracted ion chromatograms (XIC) for the diagnostic m/z 204.09 ion revealed significant shifts in IgG glycosylation over the infection timeline, with samples from 36 hours post-infection dominated by EndoS-truncated IgG glycoforms (Figure 6(e)). Additionally, MS1 precursor signal quantification in combination with biodiversity analysis using the Simpson diversity index showed a progressive homogenization of IgG glycan patterns across all subclasses over time, due to increasing accumulation of truncated IgG products containing the core GlcNAc residue, with or without core fucosylation (Figure 6(d)). Fragment transitions were also generated to quantify both intact and truncated glycoforms using GlycoPOP-MS, which again revealed a time-dependent increase in truncated IgG glycoforms and a steady decrease in intact IgG levels (**Supplementary data 11**). Individual animals displayed variability in glycosylation, with some mice showing detectable IgG deglycosylation as early as 12 hours post-infection (Figure 6(f-j)). At the same time, transient increase of the levels of extended glycoform reflects hemoconcentration caused by vascular leakage during early sepsis, which increases the relative plasma protein content in the fixed sample volume used for analysis. Interestingly, we also observed a reduction in both intact and truncated IgG3 levels. These results are consistent with previous findings in this model of a secondary proteolytic mechanism mediated by the streptococcal enzyme IdeS that primarily cleaves IgG3 leading to reduced total IgG3 levels (Figure 6(j)). Together, these results demonstrate that population-level glycoform analysis facilitates the quantification of both glycan and protein changes in samples from in vivo models, highlighting its general utility to study IgG responses in disease models, e.g., infections.

## Discussion

We present a novel experimental and computational framework to analyze protein glycosylation by leveraging spectral, biosynthetic, and physicochemical relationships across glycoform populations. This approach enables the mapping of glycosylation changes in murine and human glycoproteins in biopharmaceuticals and infection models. Specifically, the approach facilitates detailed characterization of the IgG and AT3 glycosylation landscape, capturing the broad spectrum of modifying glycans, site-specific heterogeneity, and dynamic changes in response to biosynthetic manipulation or disease states, as shown here for streptococcal infections.

Unlike conventional glycoproteomic approaches, which typically focus on individual glycoforms, our population-level analysis exploits relationships among glycoforms to enhance identification and quantification. For example, organizing glycopeptide MS data into networks based on spectral similarities improves glycoform identification. Combining these networks with algorithms such as network walks significantly expands the repertoire of detectable IgG glycoforms within a given sample, including low-abundant glycoforms, to provide a more nuanced and complete view of site-specific glycosylation.

Notably, our approach also reveals that both the IgG and AT3 glycan repertoires are consistently biased toward a few highly abundant glycoforms within a much larger population of low-abundance species. While current glycoproteomic studies frequently acknowledge the existence of glycoform heterogeneity, there remains a lack of standardized methods to quantify and compare the degree of diversity between different biological states. By adapting statistical methods from ecology, we provide a quantitative approach to measuring glycoform diversity, making it possible to compare how much more or less heterogeneity is present in different systems or after perturbation.

In this study, we show that commercial monoclonal IgG antibodies derived from heterologous expression systems have typically less degree of diversity than the biologically relevant IgG that circulates in plasma. By using various biodiversity metrics, we show that this difference is not so much at the level of how many glycans that are present (species richness), but on the abundance distribution pattern of the whole glycoform population (species evenness), with functional IgG glycoform being more evenly distributed in plasma compared to commercial products. Moreover, we also coupled biodiversity analysis with glyco-mutant geCHO cell models to directly relate glycoform diversity to biosynthetic processes. The differences in glycoform distributions across various knock-out and knock-in mutants suggest that the abundance level of specific enzymes within the glycosylation pathway imposes distinct constraints on glycan diversity. In general, our data suggests that glycoform abundance distributions are typically skewed; however, the identity of the dominant glycoforms, their distribution patterns, and biodiversity indices such as richness and evenness vary depending on the biological context. This intrinsic bias in glycoform abundance is not unique to IgG and AT3, but has been observed across most glycoproteins studied to date.^7^

To further demonstrate the applicability of this biodiversity statistical framework, we also analyzed glycoengineered AT3, an important glycoprotein regulator of coagulation, modified with multiple glycosylation sites. This analysis revealed that the WVSNKTEGR site exhibited a broader glycoform repertoire and higher glycan diversity compared to the SLTFNETYQDISELVYGAK site, as reflected by both richness and evenness indices. This suggests that glycan biosynthesis is influenced not just by the availability of cellular enzymes and substrates, but also by site-specific constraints, such as protein structure and glycosylation site accessibility. In contrast to proteins, glycans are not explicitly encoded in the genome; however, genomic sequences influence the range of glycans that can be produced at a given location on a protein, as it determines the structure and accessibility of glycoprotein glycosylation sites.^44,45^ The abundance of each feasible glycan structure is also influenced by the regulation of various components of the glycosylation machinery (e.g., glyco-enzymes, sugar nucleotides, and chaperones), their substrate accessibility, and metabolic control within the cell. We envision that our population-level analysis could help deconvolute how these other mechanisms shape glycoform diversity in specific biological contexts by providing a more quantitative framework to assess site-specific glycan diversity.

Previous studies have shown that glycopeptides from site-specific glycoforms often co-elute in reversed-phase chromatography due to their shared peptide backbones.^15^ To address this limitation, glycan-focused separation methods such as hydrophilic interaction chromatography (HILIC) have been developed.^46^ However, these methods still primarily focus on analyzing individual glycoforms, followed by a post hoc assessment of site-specific glycan features, such as the quantification of specific monosaccharides at particular glycosylation sites. Importantly, it is often not the abundance of individual glycoforms per se, but the availability of specific glycan features within a glycoform population that holds biological relevance. For instance, the absence of core fucosylation enhances IgG Fc binding to FcγRIIa, leading to increased inflammation in contexts such as Dengue and SARS-CoV-2 virus infections.^47–49^ Similarly, deficits in IgG Fc galactosylation are linked to autoimmune diseases,^50^ while increased sialylation mediates anti-inflammatory responses.^51^

As an alternative, we developed GlycoPOP-MS, a DIA-MS approach that uses large isolation windows to directly capture co-eluting glycopeptides sharing identical peptide backbones, but differing in glycan structures. This method enhances sensitivity by detecting common glycopeptide features shared by multiple glycoforms, enabling direct quantification of glycan traits within glycoform populations. Building on previous studies that used large isolation windows to measure glycan oxonium ions,^52,53^ we extended the analysis to include other fragment ions and introduced an automated workflow for spectral library generation compatible with tools like Skyline. GlycoPOP-MS acquires both MS1 and MS2 data, and offers enhanced robustness in glycoform quantification by leveraging shorter cycle times and increased data acquisition points across chromatographic peaks, leading to more precise and reproducible measurements. Additionally, by capturing glycoform populations rather than individual species, GlycoPOP-MS provides a more comprehensive and high-throughput approach to studying glycan alterations in complex biological samples. Finally, we leveraged GlycoPOP-MS to quantify deglycosylated IgG glycopeptides in plasma from GAS infected mice, highlighting its potential to study glycosidase-mediated glycan alterations in IgG and other immune effector glycoproteins.

While GlycoPOP-MS effectively quantifies population-level glycan traits, its application to more complex mixtures of glycoproteins, with multiple glycosylation sites and more extensive microheterogeneity, might present additional challenges, primarily due to potential co-elution of different glycoform populations. To mitigate this problem, additional separation strategies could be incorporated, including complementary chromatography techniques (e.g., HILIC) to enhance the resolution of co-eluting glycoforms, gas-phase fractionation to minimize spectral complexity and improve signal-to-noise ratios, and ion mobility spectrometry to provide an additional layer of separation based on glycopeptide conformation, helping to distinguish isomeric or closely related glycoforms. Future studies integrating these approaches could further refine GlycoPOP-MS, making it more adaptable for highly complex glycoprotein mixtures while preserving its strengths in sensitivity and quantification.

Looking ahead, the versatility of our population-level analysis opens exciting opportunities to extend its application beyond IgG and AT3 to other classes of glycoproteins, including both N- and O-linked glycoproteins and those with multiple glycosylation sites, where glycoform complexity is even greater. Incorporating machine learning algorithms could further enhance its utility by uncovering features of glycan diversity that are critical in various disease contexts, providing new biomarkers or therapeutic targets. Other population-level relationships could also be exploited to refine these analyses. Notably, recent studies leverage similar principles – using spectral similarities of peptide backbone ions or chromatographic relationships – to improve glycopeptide analysis.^22,54^ The growing number of such approaches suggests an emerging paradigm shift in glycoproteomics, where multiple molecular relationships, including yet unexplored ones, can be systematically harnessed to enhance both identification and quantification. More broadly, the shift from analyzing glycoproteins as isolated entities to viewing them as families of molecules aligns with emerging perspectives in proteomics. This paradigm could be translated to the study of proteoforms,^55,56^ where the interplay of other PTMs, such as phosphorylation, acetylation, and SUMOylation, shape the structure and function of the human proteome. Following the sequencing of the human genome and the near-complete mapping of its proteome, charting these variations represents the next frontier in our quest for a deeper understanding of the regulation of molecular diversity and its implications for health and disease.

## Material and methods

### Commercial antibodies

Trastuzumab (Herceptin, HER) (Roche), Bevacizumab (Avastin, AVA) (Roche), Omalizumab (Xolair, XOL) (Novartis), Cetuximab (Erbitux, ERB) (Merck), and intravenous immunoglobulin G (IVIG) (Octagam).

### Glycoengineered antibody production and purification

Glycosyltransferase knockouts and knock-ins for the CHO cells expressing the IgG1 subclass of trastuzumab (Herceptin, HER) or human antithrombin 3 (AT3) were conducted at the Denmark Technical University. These cell lines were derived from the CHO-S cell line (Gibco Cat. # A11557-01), and they were generated and verified according to the procedures described previously(28). In addition to evaluating HER produced from CHO-S cells, in this study we engineered cells with the following combinations of modifications (1) knockout of B4galt1/2/3, (2) knockout of Fut8 and Sppl3, and (3) knockout of St3gal3/4/6, B3gnt2, Sppl3; with knock-in of human ST6GAL1. To generate each mutant line, the cells were cultured in CD CHO medium (Gibco 10,743–029) supplemented with 8 mM l-glutamine (Lonza BE17-605F) and 2 mL/L of anti-clumping agent (Gibco 0010057AE) according to the Gibco guidelines. The day prior to transfection, cells were washed and cultured in exponential phase in a medium not supplemented with anti-clumping agent. At the day of transfection, viable cell density was adjusted to 800,000 cells/mL in 125 mL shake flasks (Corning 431,143) containing 30 mL medium only supplemented with 8 mM l-glutamine. Plasmids encoding for HER or AT3, were used for transient transfections. For each transfection, 30 μg plasmid was diluted in OptiPro SFM (Gibco 12,309,019) to a final volume of 750 uL. Separately, 90 uL FuGene HD reagent (Promega E2311) was diluted in 660 uL OptiPro SFM. The plasmid/OptiPro SFM mixture was added to the FuGENE HD/OptiPro SFM mixture and incubated at room temperature for 5 min. The resultant 1.5 mL plasmid/lipid mixture was added dropwise to the cells. Supernatants containing model protein were harvested after 72 h by centrifugation of cell culture at 1000 g for 10 min and stored at − 80°C until purification and N-glycan analysis.

### Bacteria and culture conditions

Streptococcus pyogenes AP1 (from the Collection of the World Health Organization Collaborating Center for Reference and Research on Streptococci, Prague, Czech Republic) were grown in Todd – Hewitt broth, supplemented with 0.2% yeast extract (THY, BD diagnostic), overnight (o/n) at 37°C and 5% CO2 as previously reported.^43^

### Bacterial infections

All animal use and procedures were approved by the local Malmö/Lund Institutional Animal Care and Use Committee, ethical permit number 03681–2019. *S. pyogenes* strain AP1 was grown to logarithmic phase in THY medium (37°C, 5% CO2). Bacteria were washed and resuspended in sterile phosphate-buffered saline (PBS). Nine-week-old female C57BL/6J mice (Janvier, Le Genest-Saint-Isle, France) were infected with 50 µl (2×105 cfu) bacteria by subcutaneous injection on the right flank. Control groups were similarly injected with sterile saline. Bodyweight and general symptoms of infection were monitored regularly. Mice were sacrificed at 12 h, 24 h and 36 h postinfection. Blood was collected by cardiac puncture using tubes containing sodium citrate (MiniCollect tube, Greiner Bio-One).

### IgG pulldowns

IgGs were purified using the Protein G AssayMAP Bravo (Agilent) technology, according to the manufacturer’s instructions. Briefly, 10 µl plasma was diluted with PBS to a final volume of 100 µl and applied to pre-equilibrated Protein G columns. Columns were washed with PBS and eluted in 0.1 M glycine (pH2). The final pH was neutralized with 1 M Tris and saved until further use.

### Trypsin digestion and peptide desalting

Roughly ~10 µg of each commercial and glycoengineered monoclonal antibody and ~20 µg total IgG purified from mouse plasma were diluted in 8 M urea, reduced with 5 mM Tris(2-carboxyethyl)phosphine hydrochloride, pH 7.0 for 45 min at 37°C, and alkylated with 25 mM iodoacetamide (Sigma) for 30 min at room temperature, followed by dilution with 100 mM ammonium bicarbonate to a final urea concentration below 1.5 M. In a separate experiment, IVIG was preincubated with β1–3,4 galactosidase or α2–3,6,8 neuraminidase (New England Biolabs), according to manufacturer’s guidelines, before reduction and alkylation. Proteins were finally digested by incubation with trypsin (1/100, w/w, Sequencing Grade Modified Trypsin, Porcine; Promega) for at least 9 h at 37°C. Digestion was stopped using 10% trifluoracetic acid (Sigma) to pH 2 to 3. Peptide clean-up was performed by C18 reversed-phase spin columns according to manufacturer instructions (Silica C18 300 Å Columns; Harvard Apparatus). Samples were lyophilized using a vacuum concentrator (Genevac, miVac) and resuspended in 2% acetonitrile and 0.2% formic acid (Sigma) before MS analysis.

### LC-MS/MS analysis of IgG glycopeptides

IgG glycopeptides were analyzed on a Q Exactive HF-X mass spectrometer (Thermo Fisher Scientific) connected to an EASY-nLC 1200 ultra-HPLC system (Thermo Fisher Scientific), as previously reported(43). Briefly, peptides were trapped on a precolumn (PepMap100 C18 3 μm; 75 μm × 2 cm; Thermo Fisher Scientific) and separated on an EASY-Spray column (Thermo Fisher Scientific). Mobile phases of solvent A (0.1% formic acid), and solvent B (0.1% formic acid, 80% acetonitrile) were used to run a linear gradient from 4 to 45% over 60 min. MS scans were acquired in data-dependent mode with the following settings, 60,000 resolution @ m/z 400, scan range m/z 600–1800, maximum injection time of 200 ms, stepped normalized collision energy (SNCE) of 15% and 35%, isolation window of 3.0 m/z, data-dependent HCD-MS/MS was performed for the ten most intense precursor ions. For the GlycoPOP-MS workflow, the MS scans were acquired in DIA mode using: one full MS scan, followed by two consecutive MS/MS scans using window sizes of 600 Da; or one full MS scan, followed by 6 consecutive MS/MS scans using window sizes of 200 Da; or one full MS scan, followed by 40 consecutive MS/MS scans using window sizes of 30 Da. The NCE was set to either 15%, 25% or 35%, each individual or as SNCE. Maximum injection time of 200 ms, loop count of 10, and an AGC target of 1e6.

### Glycoproteomics data analysis

Raw files were searched in Byonic (Protein Metrics Inc, v5.0.3) integrated as a node in Proteome Discoverer (Thermo, v.2.5.0.400). Files were searched against a UniProt human and mouse protein sequence database using the default search strategy: enzyme: trypsin with a maximum of two missed tryptic cleavages per peptide, up to one glycan per peptide as a ‘rare’ variable modification, up to 10/20 ppm deviation of the observed precursor/product ion masses from the expected values, up to one Met oxidation (+15.994 Da) per peptide (variable ‘common’ modification), together with a predefined default glycan database of human (134 unique glycans) and mouse (83 unique glycans) structures (**Supplementary table 1**) and a decoy and contaminant database available in Byonic. Identifications with a Byonic score > 100 were considered positive hits, and their spectra were manually validated(29).

### Molecular networking

Raw spectra files were processed to MS/MS peak lists (.mgf files) using MSConvert with the continuous wavelet transformation method for peak selection. Peak lists were filtered based on the presence of the HexNAc-specific oxonium ion m/z 204.09 to retain glycopeptide scans. All glycopeptide scans were then combined and analyzed separately for each species, (i.e., human and mouse). A total of 40 human IgG DDA samples and 51 mouse IgG DDA samples from various biological contexts were considered for analysis. Molecular networks were generated using the online molecular networking workflow, METABOLOMICS-SNETS-V2, at the Global Natural Products Social Molecular Networking Server (http://gnps.ucsd.edu/). The Precursor Ion Mass Tolerance and Fragment Ion mass Tolerance used for consensus spectrum creation were set to 0.02 Da. The minimum cosine score between pairs of consensus spectrum to create a network edge was 0.7 and the minimum number of matched fragment ions to maintain that edge was set to 6. Network visualization and subsequent analysis were done in Cytoscape.^57^ Node and edge lists were further analyzed using the python workflow described below: “Network walk.”

### Network walk for annotation propagation

After initial identification of glycopeptides search using Byonic’s Glycopeptide Search, results files were used for seed annotation of the molecular networks inspired by our previous work on SweetNET(32). This seed matching had an absolute mass tolerance of 0.1 Da and allowed for the addition or removal of a single H+ ion that may appear during initial ionization of the glycopeptide. Then the propagation algorithm searches for unannotated nodes that are connected to annotated nodes and exhibit a precursor mass difference that is within 0.1 Da of the annotated node mass +/- the mass of a list of single or combined monosaccharide differences. For example, a mass difference of +15.995 could define a transferred glycopeptide annotation that has one less fucose (−146.058) and one more hexose (+162.053). The algorithm allows the user to specify the maximum possible number of changes that are allowed; for this work this was set to 2. Additionally, proximal nodes that are exactly 1 Da difference had exact glycopeptide annotation transferred to account for ionization differences (1 H+). The propagation algorithm iterates over newly annotated nodes until there are no longer any annotation transfers and the network walk is complete.

### Precursor library and hybrid spectral library generation

From the annotated molecular networks, glycopeptide hits were specified as transition lists. First, all possible ionization states of the precursors between +1 and +4 were considered. The list was also curated using N-linked glycan biosynthesis reaction rules.^34,58^ Additionally, glycopeptide hits from the molecular networks were used to generate feature-centric spectral libraries for the GlycoPOP-MS analysis. To generate an entry in the spectral library, all glycoforms observed are collected and organized into a substructure network using GlyCompare.^34,59^ This substructure network is an acyclic graph that connects nodes via an edge if the child node is a compositional substructure of the parent. For example, the galactosylated node HexNAc(4)Hex(5)Fuc(1) is a child of the sialylated parent node HexNAc(4)Hex(5)Fuc(1)NeuAc(1). Using the annotated glycoforms as a starting point, GlyCompare infers each compositional substructure required to create the full network. Therefore, this network can represent any theoretical glycopeptide fragmentation pattern. In this way, an entire theoretical spectral library is made.

We further annotated this spectral library to contain entries that represent major glycan features, such as fucosylation, galactosylation, sialylation, and high-mannose structures, to extract intensity information from the GlycoPOP-MS raw data. The user defines the criteria that a fragment ion must contain to be considered for the quantification of the glycan feature. For example, fucosylation must be quantitated by fragments that contain one or more fucose monosaccharides. Then the spectral library entries are searched for all fragment ions, which come from many precursor ions, that meet the defined criteria and are collapsed into a superclass precursor ion representing the defined feature. Thus, an entry in the feature-centric spectral library contains all observable oxonium and glycopeptide fragment ions that can appear for precursors in the superclass.

### Skyline feature extraction and quantification analysis

The resulting precursor and feature-centric spectral library were designed to be compatible with the open-source MS data analysis software Skyline.^60^ Once the library is loaded, known explicit retention time windows are defined for each feature. Skyline was used in molecule mode. Precursor transition settings were set to: min m/z: 100; max m/z: 1800; MS1 filtering included 3 isotope peaks and a mass accuracy of 10 ppm. Only scans within 5 min of MS/MS IDs were allowed. For the GlycoPOP-MS feature extraction the setting were as follows: MS1 filtering using 3 isotope peaks; MS/MS filtering was set to DIA, isolation scheme to 30 or 600. After results are imported into Skyline, peak picking adjustments are made to synchronize the integration of peaks across selected explicit retention time windows for each feature. Transition results are exported and prepared for feature quantification using the MSstats R package. Only transitions with feature specificity are used in feature quantification. To identify glycan-sensitive transitions for the analysis of GlycoPOP-MS data, we performed Sparse Partial Least Squares – Discriminant Analysis (PLS-DA) using the mixOmics R package.^61^ The analysis was conducted using the first two principal components (ncomp = 2) and selecting the top 10 variables per component (keepX = 10,10). The peak area for a transition found in the two 600 Da windows is summed. MSstats summarization is run without imputation with “globalStandards” normalization. The glycopeptide fragment Y1 (peptide + HexNAc(1)) was used for normalization. Differential feature abundance analysis was performed using MSstats groupComparisons method.

### Biodiversity statistical analysis

The intensity of each identified glycopeptide was extracted via Skyline, and expressed as a percentage of the total glycopeptide intensity for a particular glycosylation site. Both glycopeptide counts (e.g., how many glycoforms that were identified) and percentages were used as input to plot the species frequency/abundance distributions, and to calculate the biodiversity indices: richness, evenness, inverse Simpson index, and to draw Renyi plots. All biodiversity analysis was conducted using the functions of the Vegan R package.^62^

## Supplementary Material

Supplementary data 4.xlsx

Supplemental figures and legends_R1.pdf

Supplementary data 9.xlsx

Supplementary data 5.xlsx

Supplementary data 3.xlsx

Supplementary data 7.xlsx

Supplementary data 6.xlsx

Supplementary data 2.xlsx

Supplementary data 10.xlsx

Supplementary data 8.xlsx

Supplementary data 1.txt

## Data Availability

All mass spectrometry data associated with this manuscript has been deposited in the MassIVE repository (**MSV000096637**, password for reviewers: uqyCR4e2moIK78oB) and will be made publicly available upon publication.
