# Supplementary material for: Population-level analysis of glycoprotein glycoforms
Source: MAbs. 2026 Apr 30;18(1):2665879. doi: 10.1080/19420862.2026.2665879 (PMC13134397; doi:10.1080/19420862.2026.2665879)
Supplement: Supplemental figures and legends_R1.pdf [file KMAB_A_2665879_SM3661.pdf]

Population-level analysis of glycoprotein glycoforms

Alejandro Gomez Toledo[1], James T Sorrentino[2], Sanne Schoffelen[3], Bjørn Voldborg[3], Erika Velasquez[4], Aaron M. Scott[1], Göran Larson[5,6], Nathan E Lewis[7,8,9,10], Johan Malmström[1]

Affiliation

[1]Division of Infection Medicine, Department of Clinical Sciences, Lund University, Lund, Sweden

[2]Bioinformatics and Systems Biology Graduate Program, University of California, San Diego, La Jolla, CA, USA

[3]Department of Biotechnology and Biomedicine, Technical University of Denmark, DK-2800, Kongens Lyngby, Denmark

[4]iPSC Laboratory for CNS Disease Modelling, Department of Experimental Medical Science, BMC D10, Lund University, Lund, Sweden

[5]Department of Laboratory Medicine, Institute of Biomedicine, University of Gothenburg, Sahlgrenska University Hospital, Gothenburg, Sweden.

[6]Department of Clinical Chemistry, Region Västra Götaland, Sahlgrenska University Hospital, Gothenburg, Sweden.

[7]Departments of Pediatrics and Bioengineering, University of California, San Diego, La Jolla, CA, USA

[8]Complex Carbohydrate Research Center, University of Georgia, Athens, Georgia, USA.

[9]Department of Biochemistry and Molecular Biology, University of Georgia, Athens, Georgia, USA.

[10]Center for Molecular Medicine, University of Georgia, Athens, Georgia, USA.

Keywords: Immunoglobulin, glycosylation, glycoproteomics, glycan heterogeneity

This PDF file includes:

Supplemental figures and supplemental legends

Corresponding author: Johan Malmström

Email: johan.malmstrom@med.lu.se

Supplementary Fig. 1. General overview of the analytical pipeline based on population-level analysis developed in this study. Mouse IgG and human IgG and antithrombin 3 (AT3) from various sources were analyzed via regular glycoproteomics to generate a spectral library that could be used to quantify glycoforms. Samples were then analyzed via molecular networking in combination with a network walk algorithm, and in parallel via the GlycoPOP-MS workflow. Both glycoform diversity and glycan features were finally measured.

Supplementary Fig. 2. Assesing overlap beween glycan identification and their diversity modeling. (a) Overlap of the IgG glycopeptide identifications through the initial Byonic searches alone or together with the networking algorithm. A set of new 11 structures that were not included in the glycan databases were also identified through the networking algorithm. (b) Species frequency/abundance distributions across all quantified IgG glycoforms for the five commercial monoclonal antibodies, modeled and fitted to two different ecological statistical models of biodiversity: broken stick and pre-emption. AIC: Aikake information criterion, BIC: Bayesian information criterion.

Supplementary Fig. 3. Optimization of selection of transitions for the GlycoPOP-MS pipeline. (a) Quantification of the ion intensity associated with m/z 204.09 from IgG1 glycopeptides in IVIG in relation to isolation window size. **(b)** Intensity of selected oxonium ion and glycopeptide ion transitions in relation to both isolation window and collisional energy. **(c)** Partial least square discriminant analysis (PLS-DA) of AVA and IVIG samples based on combination of specific analytical features: transition type, isolation window and collisional energy. Top 20 contributing features to separation along component 1 **(d)** and component 2 **(e)**. Transitions that are informative of galactose content are highlighted in yellow, those of sialic acids are colored in magenta, and high-mannose in green.

Supplementary Fig. 4. Glycosidase treatments of IVIG samples coupled to GlycoPOP-MS analysis. Chromatographic profiles of IgG1 glycopeptides from (a) untreated IVIG samples or pretreated with (b) galactosidases or (c) sialidases followed by GlycoPOP-MS analysis. The MS1 profiles of co-eluting neutral glycopeptide show a decrease in the abundance of galactosylated precursors between (d) untreated and (e) galactosidase treated samples. The difference in precursor galactosylation is recapitulated at the level of galactosylated oxonium transition (e.g., m/z 366.14) in (f) untreated vs (g) enzyme treated samples, as well as in galactosylated glycopeptide transitions (e.g., m/z 1398.55, m/z 1479.58) in (h) untreated vs (i) enzyme treated samples. The MS1 profiles of co-eluting sialylated glycopeptides show a decrease in the abundance of precursors capped with sialic acids between (j) untreated and (k) sialidase treated IVIG samples. The difference in precursor

sialylation is recapitulated at the level of sialylated oxonium transition (e.g., m/z 292.10, m/z 657.24) in (l) untreated vs (m) enzyme treated samples.

Supplementary Fig. 5. GlycoPOP-MS results in more robust MS1 glycopeptide quantification compared to a standard DDA LC-MS/MS workflow. On top of DIA fragmentation using broad isolation windows (600Da), GlycoPOP-MS also acquires MS1 precursor information. GlycoPOP-MS acquires more data point across chromatographic peaks for both (a) AVA and (b) IVIG compared to a standard DDA LC-MS/MS workflow. Analysis of the MS1 precursor signal associated with IgG1 glycopeptides carrying the HexNAc(4)Hex(3)Fuc(1) glycan structure shows that, compared to a DDA workflow, GlycoPOP-MS result in the acquisition of more points across peaks due to significantly shorter cycle time, without affecting the total area of the chromatographic peak, which translates in lower coefficient of variations and therefore more robust quantification for both (c) AVA and (d) IVIG samples.

Supplementary data 1. List of glycan structures used for the initial Byonic searches of human and murine glycopeptides.

Supplementary data 2. Byonic search result for human IgG samples.

Supplementary data 3. Molecular networking of human IgG glycopeptides.

Supplementary data 4. Human IgG theoretical network build through GlyCompare and annotated with the experimentally identified IgG glycoforms.

Supplementary data 5. Precursor ion quantification of IgG monoclonal antibodies using Skyline.

Supplementary data 6. Byonic search results for human antithrombin 3 (AT3) samples.

Supplementary data 7. Molecular networking of human antithrombin 3 (AT3) glycopeptides.

Supplementary data 8. Extracted features and quantification of IVIG and AVA glycopeptides using different energies and isolation windows for optimization of GlycoPOP-MS.

Supplementary data 9. GlycoPOP-MS quantification results for commercial monoclonals using Skyline.

Supplementary data 10. Molecular networking of murine IgG glycopeptides.

Supplementary data 11. GlycoPOP-MS quantification results for murine IgG using Skyline.

Supplementary table 1. List of transitions to quantify glycan features through GlycoPOP-MS. Gal: galactosylation; High_Man: high-mannose; Sia/NeuAc: sialic acid/neuraminic acid; Quant: Transition for overall glycosylated IgG normalization.

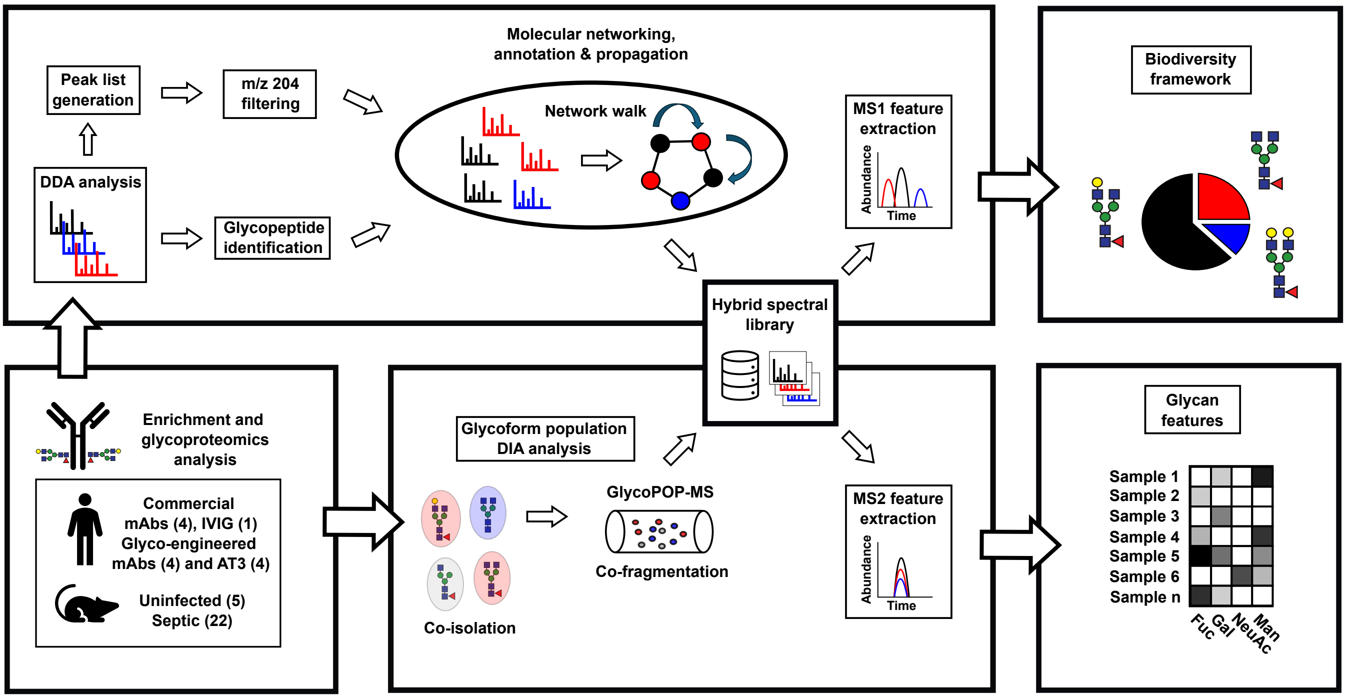

Supplementary Fig. 1. General overview of the analytical pipeline based on population-level analysis developed in this study. Mouse IgG and human IgG and antithrombin 3 (AT3) from various sources were analyzed via regular glycoproteomics to generate a spectral library that could be used to quantify glycoforms. Samples were then analyzed via molecular networking in combination with a network walk algorithm, and in parallel via the GlycoPOP-MS workflow. Both glycoform diversity and glycan features were finally measured.

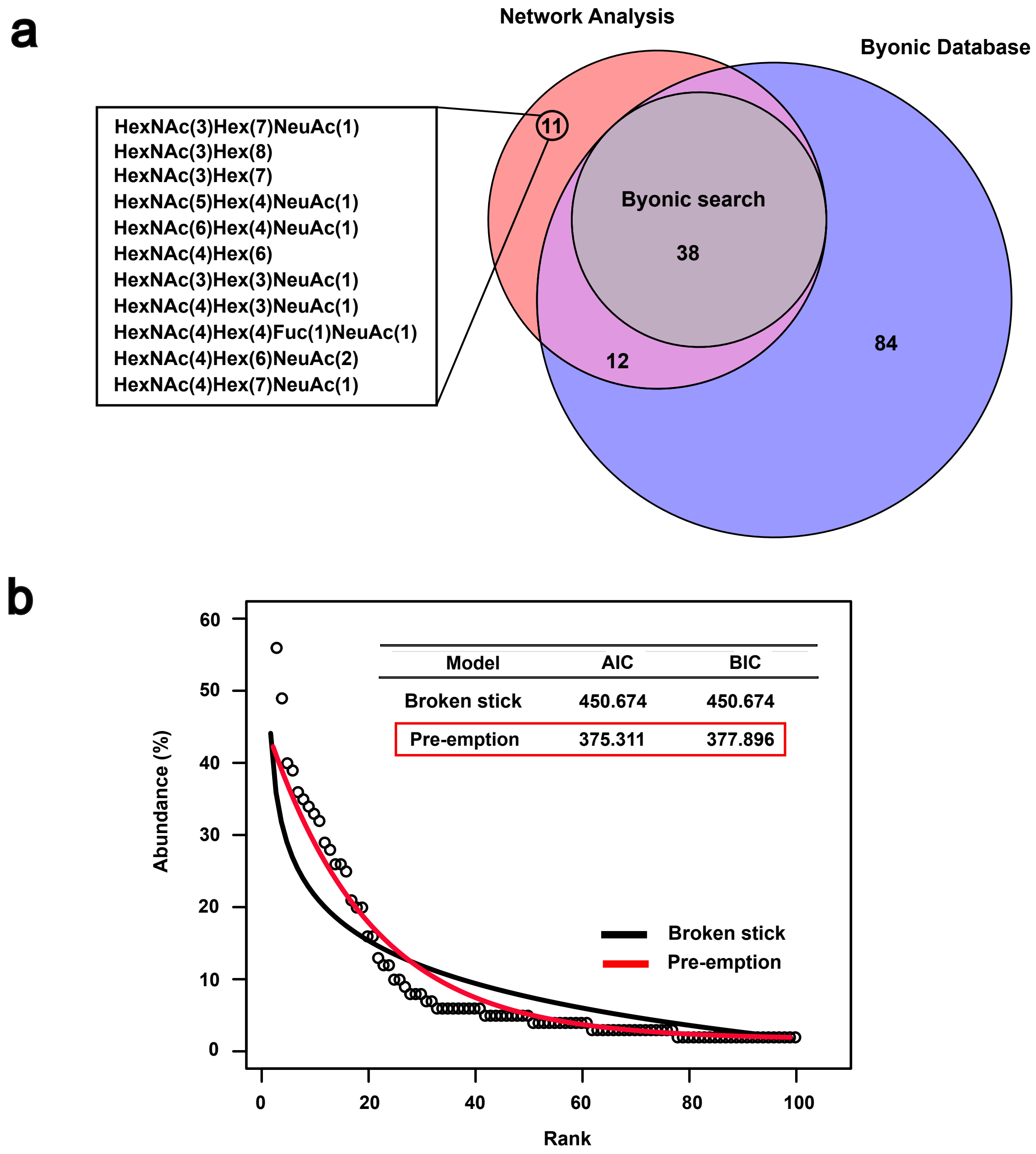

Supplementary Fig. 2. Assesing overlap beween glycan identification and their diversity modeling. (a) Overlap of the IgG glycopeptide identifications through the initial Byonic searches alone or together with the networking algorithm. A set of new 11 structures that were not included in the glycan databases were also identified through the networking algorithm. (b) Species frequency/abundance distributions across all quantified IgG glycoforms for the five commercial monoclonal antibodies, modeled and fitted to two different ecological statistical models of biodiversity: broken stick and pre-emption.
AIC: Aikake information criterion, BIC: Bayesian information criterion.

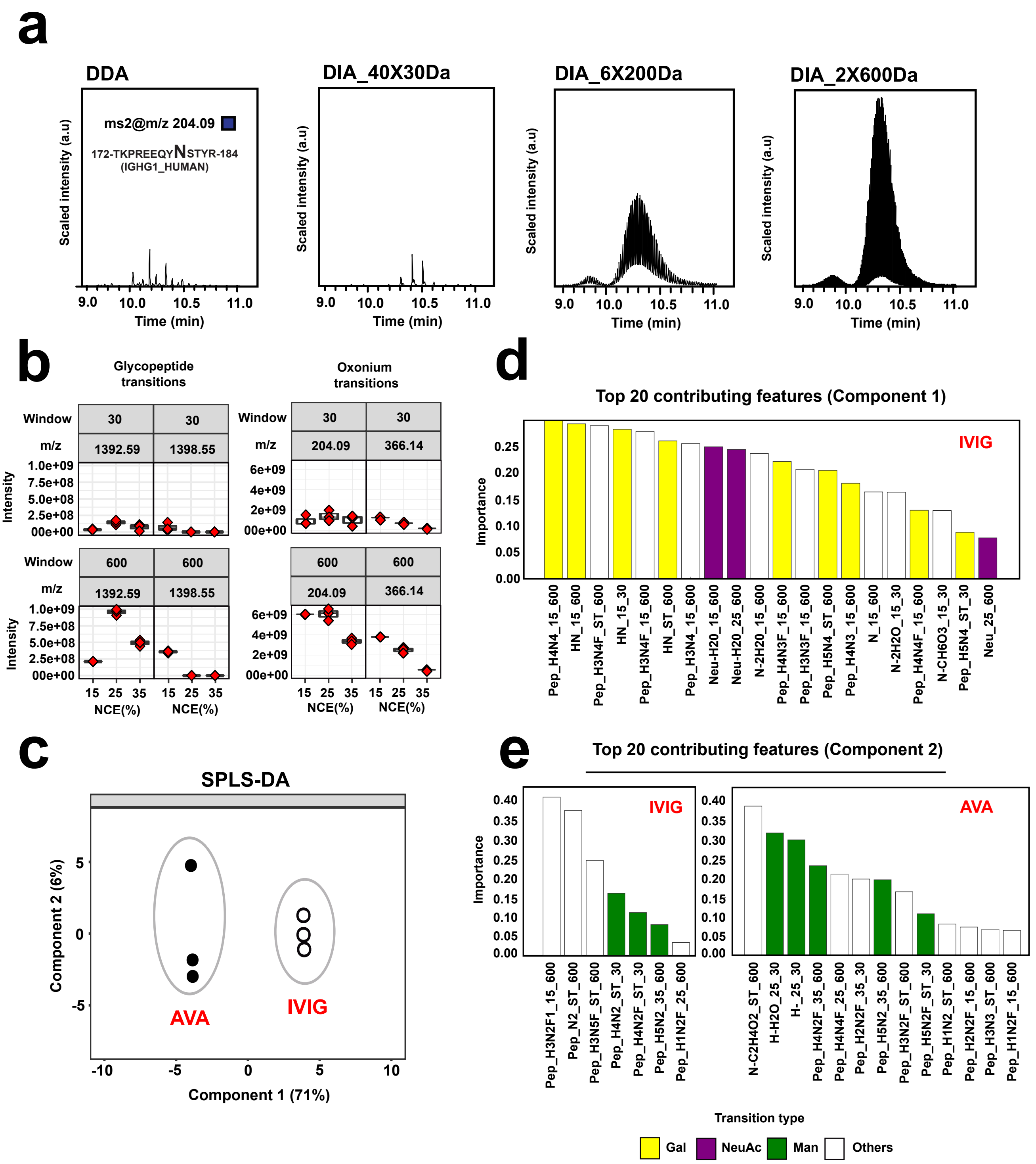

Supplementary Fig. 3. Optimization of selection of transitions for the GlycoPOP-MS pipeline. (a) Quantification of the ion intensity associated with m/z 204.09 from IgG1 glycopeptides in IVIG in relation to isolation window size. **(b)** Intensity of selected oxonium ion and glycopeptide ion transitions in relation to both isolation window and collisional energy. **(c)** Partial least square discriminant analysis (PLS-DA) of AVA and IVIG samples based on combination of specific analytical features: transition type, isolation window and collisional energy. Top 20 contributing features to separation along component 1 **(d)** and component 2 **(e)**. Transitions that are informative of galactose content are highlighted in yellow, those of sialic acids are colored in magenta, and high-mannose in green.

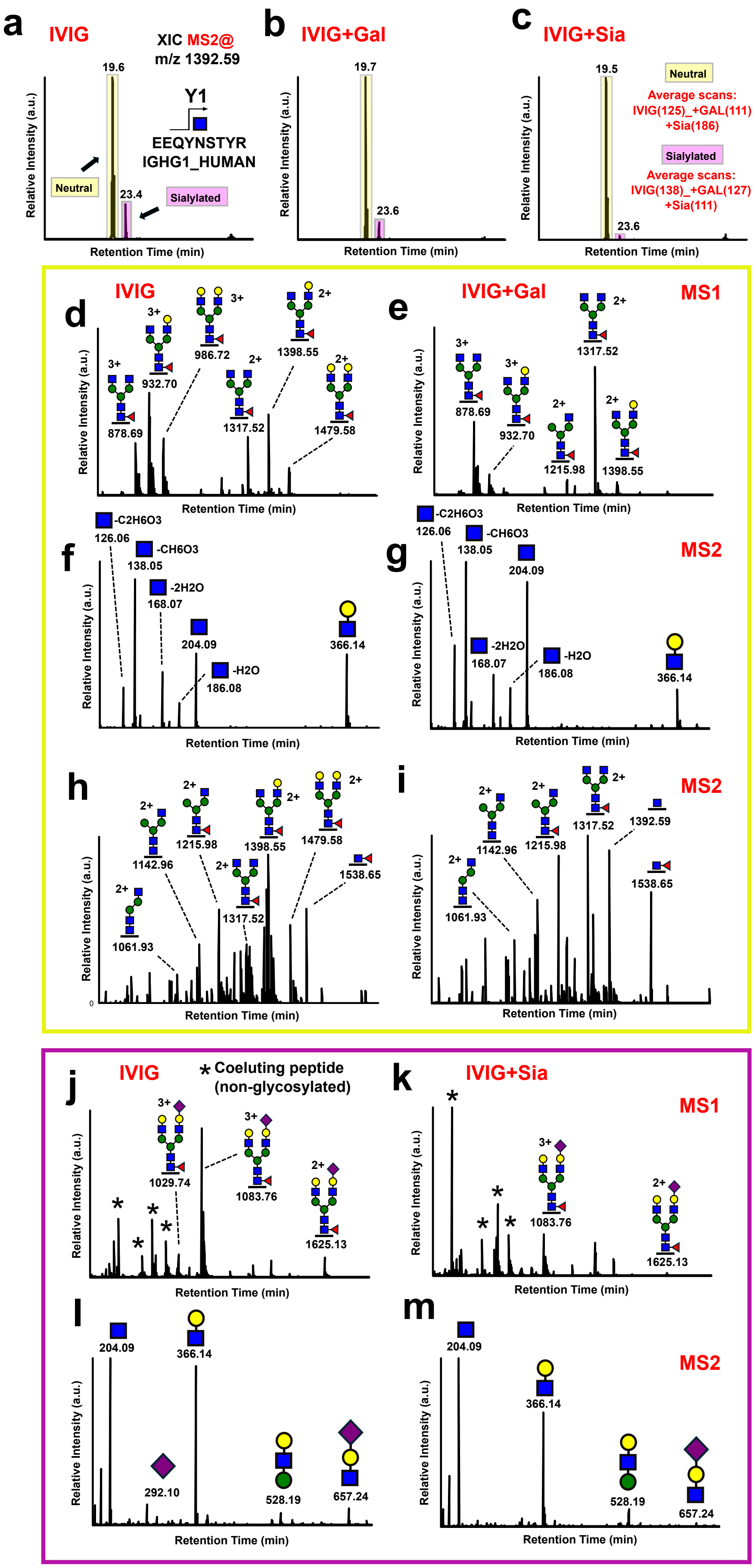

Supplementary Fig. 4. Glycosidase treatments of IVIG samples coupled to GlycoPOP-MS analysis. Chromatographic profiles of IgG1 glycopeptides from (a) untreated IVIG samples or pretreated with (b) galactosidases or (c) sialidases followed by GlycoPOP-MS analysis. The MS1 profiles of co-eluting neutral glycopeptide show a decrease in the abundance of galactosylated precursors between (d) untreated and (e) galactosidase treated samples. The difference in precursor galactosylation is recapitulated at the level of galactosylated oxonium transition (e.g., m/z 366.14) in (f) untreated vs (g) enzyme treated samples, as well as in galactosylated glycopeptide transitions (e.g., m/z 1398.55, m/z 1479.58) in (h) untreated vs (i) enzyme treated samples. The MS1 profiles of co-eluting sialylated glycopeptides show a decrease in the abundance of precursors capped with sialic acids between (j) untreated and (k) sialidase treated IVIG samples. The difference in precursor sialylation is recapitulated at the level of sialylated oxonium transition (e.g., m/z 292.10, m/z 657.24) in (l) untreated vs (m) enzyme treated samples.

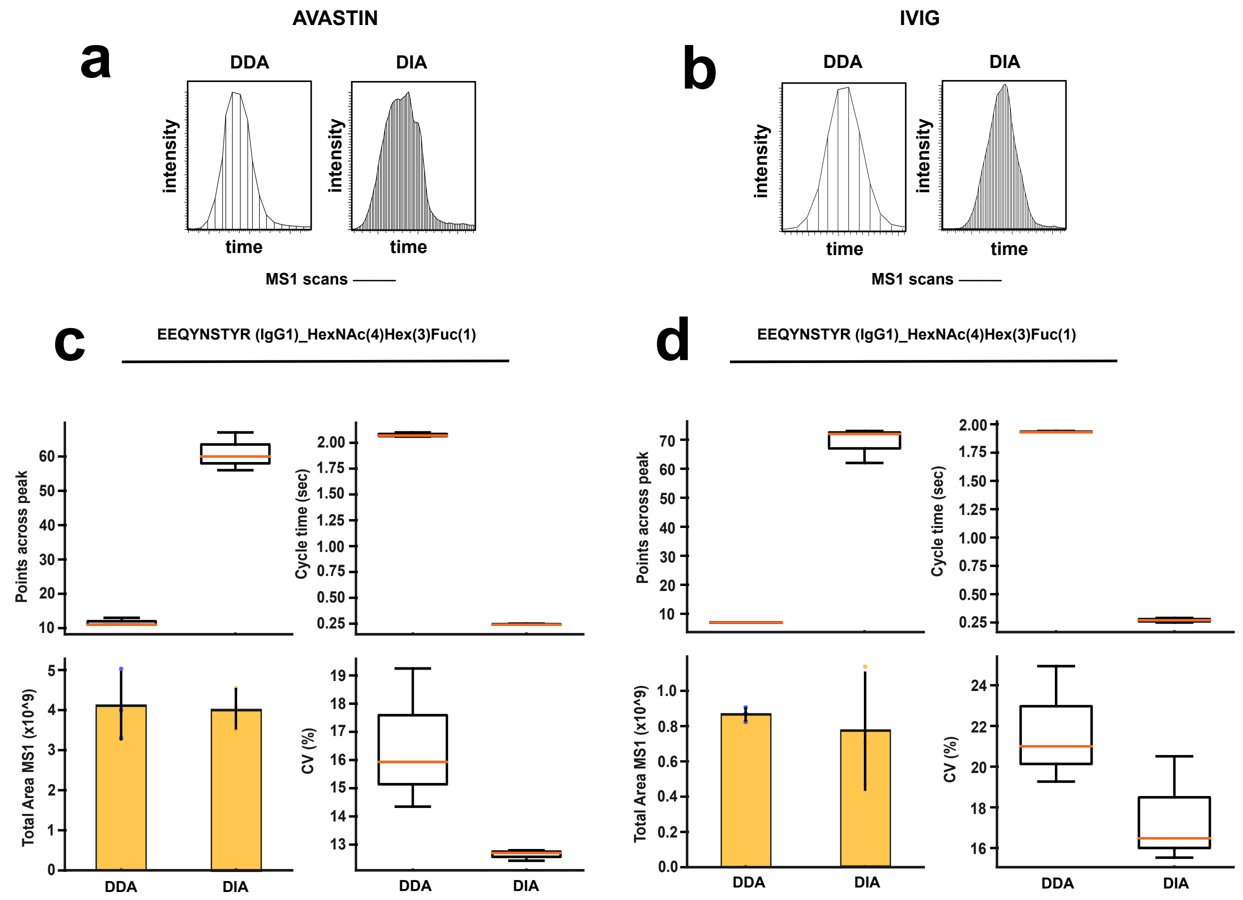

Supplementary Fig. 5. GlycoPOP-MS results in more robust MS1 glycopeptide quantification compared to a standard DDA LC-MS/MS workflow. On top of DIA fragmentation using broad isolation windows (600Da), GlycoPOP-MS also acquires MS1 precursor information. GlycoPOP-MS acquires more data point across chromatographic peaks for both (a) AVA and (b) IVIG compared to a standard DDA LC-MS/MS workflow. Analysis of the MS1 precursor signal associated with IgG1 glycopeptides carrying the HexNAc(4)Hex(3)Fuc(1) glycan structure shows that, compared to a DDA workflow, GlycoPOP-MS result in the acquisition of more points across peaks due to significantly shorter cycle time, without affecting the total area of the chromatographic peak, which translates in lower coefficient of variations and therefore more robust quantification for both (c) AVA and (d) IVIG samples.

Supplementary table 1. IgG1 GlycoPOP-MS transitions

Transition type	Transition	m/z	Charge
Gal	HexHexNAc [HN]	366.14	1
Gal	Pep_HexNAc(3)Hex(4) [Pep_H4N3]	1223.98	2
Gal	Pep_HexNAc(3)Hex(4)Fuc(1) [Pep_H4N3F]	1297.01	2
Gal	Pep_HexNAc(4)Hex(4) [Pep_H4N4]	1325.52	2
Gal	Pep_HexNAc(4)Hex(4)Fuc(1) [Pep_H4N4F]	1398.55	2
Gal	Pep_HexNAc(4)Hex(5) [Pep_H5N4]	1406.54	2
Gal	Pep_HexNAc(4)Hex(4)Fuc(1) [Pep_H5N4F]	1479.58	2
High_Man	Hex [H]	163.06	1
High_Man	Hex-H2O [H-H2O]	145.05	1
Sia	Neu5Ac [Neu]	292.10	1
Sia	Neu5Ac-H2O [Neu-H2O]	274.09	1
Sia	[NeuAc-HexHexNAc [Neu-HN]	657.24	1
Quant	Pep_HexNAc(1) [Pep_N]	696.79	2
Quant	Pep_HexNAc(1) [Pep_N]	1392.58	1
Quant	Pep_HexNAc(1)Fuc(1) [Pep_NF]	769.82	2
Quant	Pep_HexNAc(1)Fuc(1) [Pep_NF]	1538.64	1
